# Cardiovascular safety of growth hormone treatment in Noonan syndrome: real-world evidence

**DOI:** 10.1530/EC-21-0549

**Published:** 2021-12-22

**Authors:** Alicia Romano, Juan Pablo Kaski, Jovanna Dahlgren, Nicky Kelepouris, Alberto Pietropoli, Tilman R Rohrer, Michel Polak

**Affiliations:** 1 Department of Pediatrics, New York Medical College, Valhalla, New York, USA; 2 Centre for Inherited Cardiovascular Diseases, Great Ormond Street Hospital & UCL Institute of Cardiovascular Science, London, UK; 3 Department of Paediatrics, Region Västra Götaland, Sahlgrenska University Hospital, Gothenburg, Sweden; 4 US Medical Affairs, Novo Nordisk Inc., Plainsboro, New Jersey, USA; 5 Global Medical Affairs, Novo Nordisk Health Care AG, Zurich, Switzerland; 6 Department of Pediatric Endocrinology, University Children’s Hospital, Saarland University Medical Center, Homburg, Germany; 7 Paediatric Endocrinology, Diabetology and Gynaecology Department, Hôpital Universitaire Necker Enfants-Malades, AP-HP, Université de Paris, Imagine Institute, Paris, France

**Keywords:** growth hormone, hypertrophic cardiomyopathy, Noonan syndrome, pulmonary valve stenosis, real-world data

## Abstract

**Objective:**

The study aims to assess the cardiovascular safety of growth hormone (GH) treatment in patients with Noonan syndrome (NS) in clinical practice.

**Design:**

The study design involves two observational, multicentre studies (NordiNet® IOS and the ANSWER Program) evaluating the long-term effectiveness and safety of GH in >38,000 paediatric patients, of which 421 had NS.

**Methods:**

Serious adverse events, serious adverse reactions (SARs) and non-serious adverse reactions (NSARs) were reported by the treating physicians. Cardiovascular comorbidities at baseline and throughout the studies were also recorded.

**Results:**

The safety analysis set comprised 412 children with NS (29.1% females), with a mean (s.d.) baseline age of 9.29 (3.88) years, treated with an average GH dose of 0.047 (0.014) mg/kg/day during childhood. Cardiovascular comorbidities at baseline were reported in 48 (11.7%), most commonly pulmonary valve stenosis (PVS) and atrial septal defects. Overall, 22 (5.3%) patients experienced 34 safety events. The most common were the NSARs: headache (eight events in seven patients) and arthralgia (five events in three patients). Two SARs occurred in one patient (brain neoplasm and metastases to spine). No cardiovascular safety events were recorded in patients with NS. Five cardiovascular comorbidities in five patients were reported after initiation of GH treatment: three cases of unspecified cardiovascular disease, one ruptured abdominal aortic aneurysm and one PVS.

**Conclusions:**

GH treatment had a favourable safety profile in patients with NS, including those with cardiovascular comorbidities. Prospective studies are warranted to systematically assess the safety of GH treatment in patients with NS and cardiovascular disease.

## Introduction

Noonan syndrome (NS) is a genetic condition characterised by short stature, a characteristic facial appearance, skeletal anomalies and cardiovascular disease (1, 2). NS is an autosomal dominant disorder and has been associated with several genes related to the RAS/mitogen-activated protein kinase cascade (3, 4).

NS is one of the main syndromic causes of cardiovascular disease in children (5). Cardiovascular anomalies commonly associated with NS include pulmonary valve stenosis (PVS; prevalence 50–60%), hypertrophic cardiomyopathy (HCM; 20%) and atrial septal defects (6–10%) (1).

Growth hormone (GH) treatment in patients with NS improves height velocity, height standard deviation score (SDS) and adult height, with a good safety profile (6, 7). Norditropin® (Novo Nordisk A/S) is currently the only GH replacement therapy approved internationally for the treatment of short stature in children with NS (in the United States, Canada, European Union, UK, Japan, Israel, Brazil, South Korea, Switzerland and Argentina). Despite limited data showing low rates of cardiovascular events and no change in left ventricular wall thickness with GH treatment, some concerns persist about the role of GH in progression of cardiovascular disease in patients with NS (8, 9, 10, 11, 12, 13, 14, 15, 16, 17).

The objective of this study was to describe the cardiovascular safety in patients with NS treated with GH in clinical practice using data from two large observational studies.

## Materials and methods

### Study design and ethics

The NordiNet® International Outcomes Study (IOS; NCT00960128) and ANSWER Program (NCT01009905) were observational, multicentre studies monitoring the real-world long-term outcomes of GH treatment (Norditropin®, Novo Nordisk A/S) in children and adults. The studies were performed between 2002 and 2016 and involved 676 clinics in 23 countries across Europe, the Middle East and the United States (18, 19).

The patient population reported here comprises the safety analysis set (SAS) including all patients with NS under 18 years of age with at least one Norditropin® prescription recorded. The SAS included both GH-naive and non-naive patients.

Both studies were conducted with approval from relevant ethics committees. Written consent was obtained from all patients after full explanation of the purpose and nature of all procedures used, and pseudonymisation of all data was carried out in accordance with the Declaration of Helsinki, Guideline for Good Pharmacoepidemiology Practices and regulatory requirements.

### Safety assessments

Safety events were reported by treating physicians in a case report form and are defined as a collection of serious adverse events (SAEs), serious adverse reactions (SARs; considered related to GH treatment either by the reporting physician or the study sponsor) and non-serious adverse reactions (NSARs; related to GH treatment). Events that were non-serious and not related to GH treatment were not included in this report to maintain compatibility of datasets from NordiNet® IOS and ANSWER Program. Cardiovascular comorbidities reported at baseline and diagnosed throughout the duration of the studies were also recorded.

### Statistical analysis

All codes for statistical analyses were written using the SAS 9.4 software. Baseline and safety data were summarised using descriptive statistics. Statistical comparison of baseline characteristics between the SAS and patients who experienced at least one safety event was carried out with Fisher’s test for categorical variables and with *t*-test or Mann–Whitney *U* test (when the normality assumption was not met) for continuous variables. A statistically significant difference was defined as *P*  < 0.05.

## Results

### Baseline characteristics

Of the 17,995 and 20,204 paediatric patients registered in NordiNet® IOS and the ANSWER Program, respectively, 156 and 265 had NS. Of these, nine children were excluded owing to missing valid data about GH dose (missing start or stop date of GH treatment or missing dose; *n* = 7) or GH exposure (no treatment periods reported; *n* = 2). Thus, the SAS comprised 154 patients in NordiNet® IOS and 258 in the ANSWER Program (Supplementary Fig. 1, see section on supplementary materials given at the end of this article).

The combined SAS of 412 children with NS consisted of 70.9% males and 29.1% females with a mean (s.d.) age of 9.29 (3.88) years and height SDS of −2.65 (0.95) at baseline (Table 1). Mean (s.d.) baseline GH dose was 0.044 (0.014) mg/kg/day, and average GH dose during childhood was 0.047 (0.014) mg/kg/day. There were no statistically significant differences in baseline characteristics between patients in the SAS and those experiencing safety events (*n*  = 22; Table 1), with the exception of baseline height SDS (*P* = 0.0322).

**Table 1:** Baseline characteristics.

	All patients		Patients with safety events	
	Number of patients	Mean (s.d.)^a^	Number of patients	Mean (s.d.)^a^
Female/male (*n* and %)	120/292	29.1%/70.9%	9/13	40.9%/59.1%
Age (years)	412	9.3 (3.9)	22	9.7 (4.1)
Height SDS^b^	371	−2.65 (0.95)	17	−3.13 (0.79)
Weight SDS^b^	308	−2.03 (1.31)	13	−2.57 (1.54)
Bone age/chronological age	163	0.83 (0.19)	8	0.87 (0.10)
IGF1 SDS (22)	162	−1.13 (1.62)	7	−1.22 (1.98)
GH dose at baseline (mg/kg/day)	404	0.044 (0.014)	21	0.040 (0.019)
GH-naïve at baseline, yes (%)	282	68.5%	12	54.6%
Duration of treatment (years)	412	3.1 (2.6)	22	3.3 (2.5)
GH dose during childhood (mg/kg/day)	412	0.047 (0.014)	22	0.047 (0.016)

^a^Unless otherwise specified. ^b^Height and weight SDS were calculated using age- and gender-specific national references.

GH, growth hormone; IGF1, insulin-like growth factor I; SDS, standard deviation score.

The most common concomitant medications were CNS stimulants (12.4%), antihistamines (6.6%) and thyroxine replacement (5.1%) (Supplementary Table 1). Anti-hypertensive treatment was reported in four patients (1.0%) who received diuretics, of which three (0.7%) were also prescribed angiotensin-converting enzyme inhibitors. Steroid drugs were prescribed to 20 patients (4.9%).

### Genotypes

Among the 61 patients with available genotypes, 66 genetic mutations in 5 genes were observed: *PTPN11* (*n*  = 56), *RAF1* (*n*  = 5), *KRAS* (*n*  = 2), *SOS1* (*n*  = 2) and *SHOC2* (*n*  = 1). Three patients had mutations in multiple genes, all including *PTPN11*. Of these, two patients had mutations in two genes (*PTPN11* and *SOS1*; *PTPN11* and *RAF1*) and one in four genes (*PTPN11, SOS1, RAF1*and *KRAS*).

### Safety

#### Adverse events

In total, 22 (5.3%) patients with NS experienced 34 safety events (Supplementary Table 2). Adverse reactions (NSARs or SARs) occurred in 18 (4.4%) patients after the initiation of GH treatment. Of the 24 NSARs, the most common were headache (eight events in seven patients) and arthralgia (five events in three patients).

Two SARs occurred in a single patient after 2.5 years of treatment: brain neoplasm (metastatic fourth ventricular pilocytic astrocytoma) and metastases to spine, possibly related to GH. The patient (aged 15.9 years) had a mutation in the *PTPN11* gene and had a history of headaches. Both events were reported as not resolved at the end of the follow-up period.

No cardiovascular safety events were recorded in a case report form by the investigating physicians during the studies. One cerebrovascular event (moyamoya disease) was reported in one patient (female, 10.4 years) after 4.1 years of GH treatment. The event was considered serious and unlikely to be related to GH treatment.

#### Cardiovascular comorbidities

Forty-eight (11.7%) patients reported a cardiovascular comorbidity at baseline (Table 2). The most common cardiovascular comorbidities at baseline were PVS (18 patients) and atrial septal defects (4 patients). In addition, three patients had HCM at baseline.

**Table 2:** Cardiovascular comorbidities.

Diagnosis	At baseline	After GHT start
Pulmonary valve stenosis	18	1
Atrial septal defect	4	–
Cardiac murmur, unspecified	3	–
Hypertrophic cardiomyopathy	3	–
Cardiovascular disease, unspecified	1	3
Cardiac disease, unspecified	2	–
Coarctation of aorta	2	–
Ventricular septal defect	2	–
Benign and innocent cardiac murmurs	1	–
Cardiac arrest with successful resuscitation	1	–
Cardiomegaly	1	–
Cardiovascular disorder originating in the perinatal period, unspecified	1	–
Atrioventricular septal defect	1	–
Mitral valve disease, unspecified	1	–
Other pulmonary valve disorders	1	–
Aortic valve disorder, unspecified	1	–
Ruptured abdominal aortic aneurysm	–	1
Stenosis of pulmonary artery	1	–
Tetralogy of Fallot	1	–

GHT, growth hormone treatment.

Five cardiovascular comorbidities in five patients were reported after the initiation of GH treatment: three cases of unspecified cardiovascular disease, one case of PVS and one ruptured abdominal aortic aneurysm. The first four patients had no reported SAEs. The abdominal aortic aneurysm was diagnosed in a patient with a *PTPN11* mutation 2.2 years after GH treatment initiation. This patient was also diagnosed with Crohn’s disease (1.8 years after treatment initiation) and with a glioneuronal tumour (1 week after the aneurysm diagnosis).

## Discussion

Our report of GH treatment in patients with NS in a real-world setting did not reveal substantial clinically significant cardiovascular safety signals. No cardiovascular safety events were reported, which is encouraging, particularly given the pre-existing cardiovascular comorbidities in some patients. Of the 18 patients with PVS and 3 with HCM at baseline, no worsening of these conditions during treatment with GH was reported. Among the commonly used concomitant medications, most were not relevant from a cardiac perspective.

The overall number of cardiovascular comorbidities at baseline was low (11.7%), considering that up to 80% of individuals with NS have been reported to have a cardiac anomaly (5). This contrast in prevalence suggests that patients with pre-existing cardiovascular disease may have been under-represented owing to a selection bias in GH prescribing practices, and/or the conditions may have been under-reported at baseline. Thus, as active screening of participants was not required prior to enrolment in the study, the five cardiovascular comorbidities reported after GH treatment initiation may have also been pre-existing. Because of the low prevalence of reported cardiovascular disease, the findings in relation to cardiovascular safety for patients with pre-existing cardiovascular disease need to be interpreted with caution. In particular, as there was no requirement to perform or report results of echocardiograms in children with NS to monitor for development or worsening of cardiomyopathy, and as only three children had recorded HCM at baseline, these data do not readily offer guidance for treatment of children with NS and HCM. Another limitation of the study is the scarce availability of cardiovascular data (e.g. blood pressure and heart function), which may have contributed to the underreporting of cardiovascular safety events, including worsening of existing conditions. However, the real-life setting of the study can be considered a strength.

Our findings are in agreement with previous studies, reporting no clinically significant change in cardiac function in children with NS treated with GH for 1–3 years (8, 9, 14, 17). We have identified 11 studies published between 1995 and 2019 reporting adult height outcome results from 1288 patients with NS, only 16 cardiovascular adverse events were reported. Of these events, pulmonary valve-related events comprised two cases of PVS (16), two mild progressions of PVS unlikely related to GH (11) and one increased pulmonary regurgitation (15). Among hypertrophy-related events, one case of HCM (12) and one case of worsening of HCM (15) were reported. In addition, there was one case of left ventricular hypertrophy (16), two cases of mild left ventricular wall thickening within the normal limit (9) and one case of increased biventricular hypertrophy (12). Rhythm-related events included one case each of atrial fibrillation (16), mild tachycardia lasting 1 day and mild ventricular extra-systoles, from which the patient recovered (14). The remaining events involved a supravalvular aortic stenosis (12) and a successful surgical right ventricular outflow tract procedure without any complication (9).

While no cardiovascular events were reported in our study, one serious cerebrovascular event was reported (moyamoya disease). A concern about mortality due to cerebral haemorrhage has been raised previously in the SAGhE study, in adults without NS who were treated with GH during childhood (20). However, it was later shown that such concern was mainly driven by results from the French sub-cohort of SAGhE (21). Additionally, the investigating physician considered the event unlikely to be related to GH treatment.

In conclusion, the evidence suggests a favourable safety profile of GH treatment in patients with NS, including those with cardiovascular comorbidities and those receiving concomitant medications. However, the low prevalence of cardiovascular comorbidities in our patient population highlights the importance of baseline cardiovascular assessments before initiating GH therapy, particularly in patients with genotypes associated with a higher cardiovascular risk. Prospective studies are warranted to systematically assess the safety of GH treatment in patients with NS and pre-existing cardiovascular disease.

## Supplementary Material

SUPPLEMENTARY MATERIAL

## Declaration of interest

A R: consultant and speaker for Novo Nordisk; consultant for Ascendis Pharma. J P K: consultant and speaker for Novo Nordisk. J D: speaker for Novo Nordisk. N K: employee of Novo Nordisk and stockholder in Novo Nordisk and Pfizer. A P: employee of Novo Nordisk and stockholder in Novo Nordisk. T R R: consultant and speaker for Novo Nordisk. M P: Novo Nordisk growth international advisory board member and has received grants from Novo Nordisk.

## Funding

This work was supported by Novo Nordisk http://dx.doi.org/10.13039/501100004191 Health Care AG.
