# Supplementary material for: Cardiovascular safety of growth hormone treatment in Noonan syndrome: real-world evidence
Source: Endocr Connect. 2021 Dec 22;11(1):e210549. doi: 10.1530/EC-21-0549 (PMC8859970; doi:10.1530/EC-21-0549)
Supplement: SUPPLEMENTARY MATERIAL [file supplementary_material.pdf]

Cardiovascular safety of growth hormone treatment in Noonan syndrome: real-world evidence

Alicia Romano[1]*, Juan Pablo Kaski[2]*, Jovanna Dahlgren[3], Nicky Kelepouris[4], Alberto Pietropoli[5], Tilman R. Rohrer[6], Michel Polak[7]

*Shared first authorship

[1]*Department of Pediatrics, New York Medical College, Valhalla, NY, USA*

[2]*Centre for Inherited Cardiovascular Diseases, Great Ormond Street Hospital & UCL Institute of Cardiovascular Science, London, UK*

[3]*Department of Paediatrics, Region Västra Götaland, Sahlgrenska University Hospital, Gothenburg, Sweden*

[4]*US Medical Affairs, Novo Nordisk Inc., Plainsboro, NJ, USA*

[5]*Global Medical Affairs, Novo Nordisk Health Care AG, Zurich, Switzerland*

[6]*Department of Pediatric Endocrinology, University Children's Hospital, Saarland University Medical Center, Homburg, Germany*

[7]*Paediatric Endocrinology, Diabetology and Gynaecology Department, Hôpital Universitaire Necker Enfants-Malades, AP-HP, Université de Paris, Imagine Institute, Paris, France*

SUPPLEMENTARY MATERIAL

Supplementary Table 1: Concomitant medication

Concomitant medication class	Patients (n)
Central nervous system stimulant	51
Antihistamine	27
Thyroxine replacement	21
Dietary supplements	15
Vitamin D_3	14
Proton pump inhibitor	13
Selective serotonin reuptake inhibitor	11
Leukotriene inhibitor	10
Alpha2-adrenoreceptor agonist	9
Circadian hormone; non-steroidal anti-inflammatory analgesic	8
Anticonvulsant; calcium replacement	7
GnRH agonist; steroid/anti-inflammatory agent; vitamin D_2	6
Antibiotic; hormone	5
Alpha-agonist; antibiotic	4
ACE inhibitor; antipsychotic; beta2-agonist (bronchodilator); histamine 2 receptor blocker; loop diuretic; steroid/anti-inflammatory/immunosuppressant; topical steroid	3
5-HT_1 agonist; aminosalicylates; antacid (H2 blocker); anticholinergic bronchodilator; antidepressant; antispasmodic; benzodiazepine; beta blocker; cardiac glycoside; fluoride replacement; gastric motility stimulant; haemostatic agent; insulin replacement; laxative; laxative (PEG); norepinephrine re-uptake inhibitor; oestrogen; opioid analgesic; phenylpiperazine; antidepressant; anabolic steroid	2
Anti-fungal; anti-oestrogen; antimetabolite; aromatase inhibitor; central nervous system depressant; carnitine replacement; co-enzyme Q10; disaccharide (sugar); IGF-I; local anaesthetic; neurotransmitter; pituitary hormone; probiotic; steroid/anti-inflammatory; thiazide diuretic; vitamin A	1

5-HT_1, 5-hydroxytryptamine (serotonin) receptor, ACE, angiotensin-converting enzyme; GnRH, gonadotropin-releasing hormone; IGF-I, insulin-like growth factor I; PEG, polyethylene glycol.

Supplementary Table 2: Summary of safety events in patients with Noonan syndrome

System organ class	Preferred term	NSAR	SAR	SAE not related to GH	Total number of events (patients)
Cardiovascular	Any	0	0	0	0
Neoplasms	Brain neoplasm	–	1 (1)	1 (1)	2 (2)
	Metastases to spine	–	1 (1)	–	1 (1)
	Glioneuronal tumour	–	–	1 (1)	1 (1)
Musculoskeletal	Arthralgia	5 (3)	–	–	5 (3)
	Myalgia	2 (2)	–	–	2 (2)
	Scoliosis	2 (2)	–	1 (1)	3 (3)
Miscellaneous	Headache	8 (7)	–	–	8 (7)
	Giant cell epulis	–	–	1 (1)	1 (1)
	Epilepsy	–	–	1 (1)	1 (1)
	Condition aggravated	1 (1)	–	1 (1)	2 (2)
	Spinal fusion surgery	–	–	1 (1)	1 (1)
	Moyamoya disease	–	–	1 (1)	1 (1)
	Other*	6 (5)	–	–	6 (5)
	**Total**	**24 (17)**	**2 (1)**	**8 (5)**	**34 (22)**

Values are shown as number of events (number of patients). A patient may have experienced more than one event.

*Other NSARs included oedema, injection site erythema, growing pains, muscle spasms, off-label use and injection site extravasation.

GH, growth hormone; NSAR, non-serious adverse reaction; SAE, serious adverse event; SAR, serious adverse reaction.

Supplementary Figure 1: Patient disposition

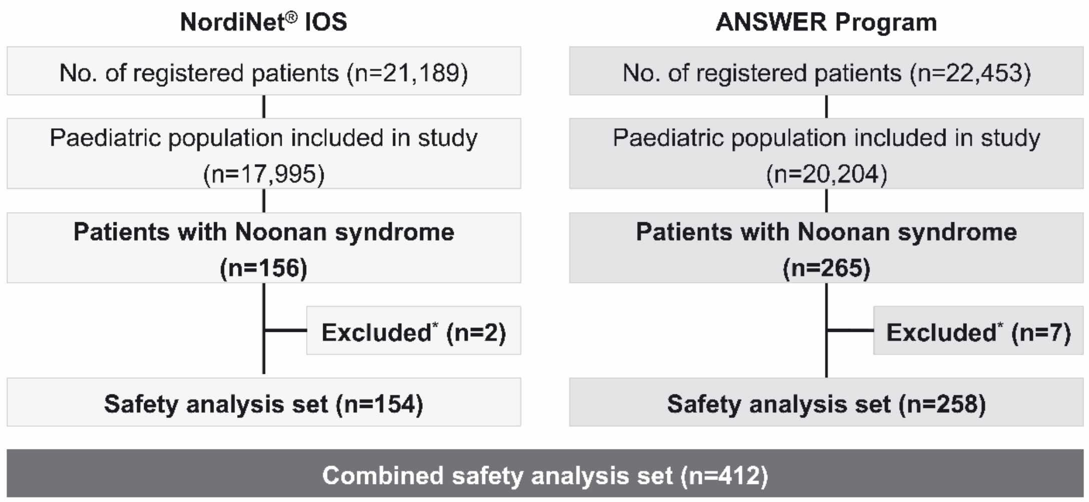

The safety analysis set includes all patients with available birthdate information who were <18 years of age and with at least one Norditropin® prescription recorded.

*Reasons for exclusion for the combined studies: no valid mean GH dose, n=7; no valid GH exposure: n=2.

IOS, international outcomes study.

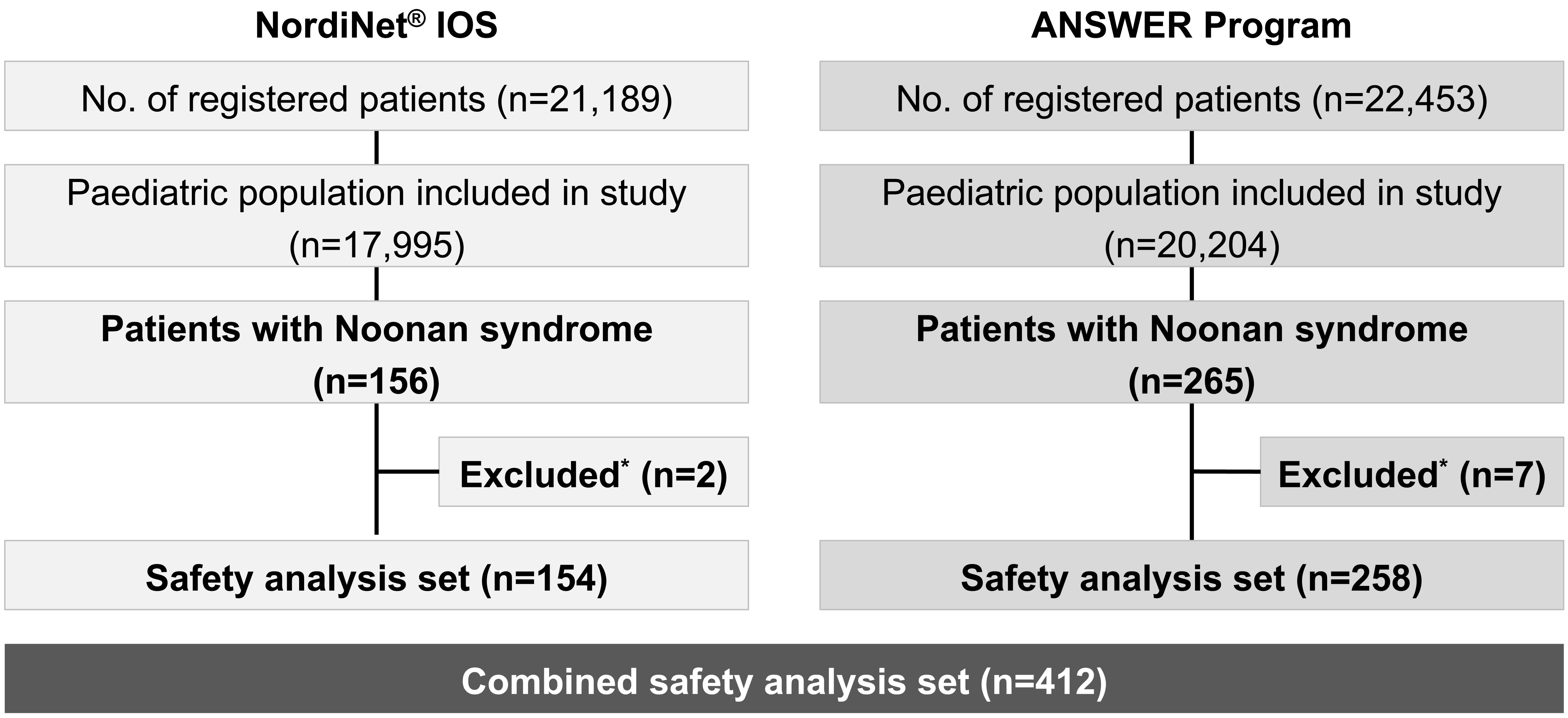
NordiNet® IOS
ANSWER Program
No. of registered patients (n=21,189)
No. of registered patients (n=22,453)
Paediatric population included in study (n=17,995)
Paediatric population included in study (n=20,204)
Patients with Noonan syndrome (n=156)
Patients with Noonan syndrome (n=265)
Excluded* (n=2)
Excluded* (n=7)
Safety analysis set (n=154)
Safety analysis set (n=258)
Combined safety analysis set (n=412)
